# Study of resilience and environmental adversity in midlife health (STREAM)

**DOI:** 10.1007/s00127-015-1126-y

**Published:** 2015-10-13

**Authors:** Eva Velthorst, Abraham Reichenberg, Jonathan Rabinowitz, Stephen Z. Levine

**Affiliations:** Department of Psychiatry, Icahn School of Medicine at Mount Sinai, 1425 Madison Avenue, New York, NY 10029 USA; Department of Psychiatry, Academic Medical Center, Amsterdam, The Netherlands; Department of Psychosis Studies, Institute of Psychiatry, King’s College London, London, UK; School of Social Work, Bar-Ilan University, Tel-Aviv, Israel; Department of Community Mental Health, University of Haifa, Haifa, Israel

**Keywords:** Common (mental) health problems, Mid-adulthood, Adversity, Resilience

## Abstract

**Purpose:**

The Jerusalem study of resilience and environmental adversity in midlife health (STREAM) was established to examine the prevalence of common mental and physical health issues in mid-adulthood in the inner city of Jerusalem, and to examine their association with lifespan psychosocial factors of vulnerability and resilience.

**Method:**

Participants were 811 randomly selected individuals from 7000 individuals who were born and grew up in inner-Jerusalem. Participants were 34–44 years old during first wave of STREAM assessment. Initial telephone surveys took place in 2007–2008 and participants were followed-up for a second survey 1 year later. Upon funding, a new wave is planned for 2017–2018. Survey topics comprised common health problems (e.g., type 2 diabetes/migraine), health markers (e.g., BMI), and psychiatric vulnerabilities (e.g., anxiety, post-traumatic stress, depressive symptoms, psychosis). Other measures included socioeconomic status, creativity, life style behavior (e.g., smoking, exercise), social contact and adaptation to change. Survey data were retrospectively merged with data of national registry sources that included adverse psychosocial factors, psychiatric and social measures assessed across all developmental stages through midlife. This includes data available on birth factors, school achievement and adjustment, cognitive and behavioral functioning during young adulthood, psychiatric hospitalizations, immigration and socioeconomic status.

**Results:**

Results on health outcomes of the first STREAM wave indicate that prevalence rates of health problems are comparable to recent World Mental Health Surveys.

**Conclusions:**

Apart from measures on adverse psychosocial factors, STREAM provides a cohort to examine resilience to developing health problems and having a poor health and functional outcome.

**Electronic supplementary material:**

The online version of this article (doi:10.1007/s00127-015-1126-y) contains supplementary material, which is available to authorized users.

## Introduction

Inner-city populations, such as inner-Jerusalem, are at increased risk of developing mental disorders and health problems during adulthood [1–5]. The primary objective of the study of resilience and environmental adversity in midlife health (STREAM) was to explore psychosocial factors that could predict unfavorable health outcome in inner cities.

STREAM is an Israeli national cohort study to actively follow-up a representative sample of adults by linking both in-depth surveys to the wealth of national registries including data on different times across the lifespan. In addition to symptom presence/absence and severity, STREAM tapped into details, such as symptom onset (e.g., “How old were you the first time you heard or saw things that others cannot see or hear?”), functional (e.g., “What is your occupation?”) and symptomatic course (e.g., “What was the longest period of time when you felt a sense of despondency, or depression, or hopelessness most hours of the day?”) and subjective experience (e.g., “How would you assess your health in general?”) of commonly experienced health problems [6].

Our goal was not only to extend existing knowledge on psychosocial factors associated with adverse outcomes in inner-city areas, but also to explore social and developmental mechanisms underlying resilience to developing health problems, mechanisms that might be key to the development of optimal interventions in high-risk populations. Although there is growing recognition of the importance of studying resilience [7], cohort studies thus far predominantly focused on factors of risk and vulnerability.

To uniquely elaborate on prior life-course research, apart from adverse psychosocial factors, STREAM therefore also included various protective factors. Measures combined provide an overview of the life-course of a wide range of psychosocial exposures and outcomes.

Internal funding from the University of Haifa, Israel, Bar-Ilan University and the Institute of Psychiatry, Kings College London, UK, supported survey data collection.

## Design

The design is a prospective cohort study, consisting of a non-stratified random sample of 7000 individuals that were born and grew up in the inner-city of Jerusalem (as defined by the city proper, and not urban or metro areas), an area chosen for its enrichment of social environmental risk factors and adverse outcomes [8, 9]. To establish the study cohort, data were extracted from administrative registries of all those attending public school in Jerusalem between 1978 and 1988 [10] and who also appeared in a birth cohort including information on all births in the greater Jerusalem area and born between 1964 and 1976 [8].

STREAM supplements these data sources by linking them to data of the mandatory Israeli Draft board assessments at age 17 and to two ‘active’ follow-up survey measures at age 34–44, including reliable subjective and objective measurements of adversity and health.

### Integrated data sources

The STREAM cohort permits linkage to a number of national registers covering measures during different developmental stages. Linkage is done using a unique personal identification number that is given all Israeli citizens at birth or immigration. Data sources that were being linked to STREAM will be summarized below:

### Data sources

#### Regional and national surveys

##### The Jerusalem Perinatal Study

The Jerusalem Perinatal Study (JPS) is a birth cohort including all 92,408 live born children in greater Jerusalem between 1964 and 1976 [8, 9].

In this study, all births to mothers in a defined geographic area were surveyed through examination of obstetric and pediatric departments, baby clinics and face-to-face interviews with mothers. Data were collected on pre-pregnancy, prenatal and birth conditions, such as pre-pregnant smoking habits of the mother [11], obstetric conditions during labor and delivery and birth weight [12]. The total JPS sample included 51.4 % males and 48.6 % females. The average age of fathers and mothers at birth was 31.5 (SD 6.8) and 27.7 (SD 5.7), respectively. Forty-three percent of the fathers and 45 % of the mothers were born in Israel. Average number of years of education was 10.2 (SD 5.4) for fathers and 9.4 years (SD 5.1) for mothers (see Harlap [9] for more detailed demographic information about the cohort). Table 1 provides information on how the demographics of JPS match the STREAM sample.

**Table 1:** Demographic characteristics

	Not in survey (A)		Wave 1 only (B)		Wave 1 and 2 (C)		Supplement 2b (D)		Statistically significant differences^c^
	M/n	SE/%	M/n	SE/%	M/n	SE/%	M/n	SE/%	
Birth year	1970.53	0.01	1970.57	0.36	1969.26	0.18	1969.31	0.18	NS
Male	46,813	(51.43)	62	(57.41)	214	(54.59)	170	(54.70)	NS
Female	44,202	(48.57)	46	(42.59)	178	(45.41)	141	(45.30)	NS
Father year of birth	1938.69	0.03	1936.02	0.86	1935.58	0.40	1935.90	0.44	A > B, C, D
Mother year of birth	1942.88	0.02	1940.31	0.69	1939.88	0.35	1939.91	0.40	A > B, C, D
Father age	31.52	0.02	32.26	0.57	33.18	0.34	32.88	0.39	NS
Mother age	27.65	0.02	28.76	0.55	28.89	0.29	28.87	0.34	NS
Native father	38,831	(42.65)	50	(46.30)	173	(44.13)	119	(38.30)	NS
Native mother	41,128	(45.17)	49	(45.37)	176	(44.90)	126	(40.50)	NS
Native paternal grandfather^a^	14,761	(16.21)	15	(13.89)	52	(13.27)	45	(14.50)	NS
Native maternal grandmother^a^	14,137	(15.53)	13	(12.04)	43	(10.97)	39	(12.50)	*χ* ^2^ = 8.30, *df* = 3, *p* < 0.05
Number of Persons living together^b^	4.19	0.02	4.90	0.53	3.84	0.19	4.00	0.15	NS
Number of rooms^b^	2.37	0.01	2.25	0.16	2.38	0.10	2.22	0.09	NS
Number of years of paternal education	10.19	0.02	10.06	0.41	11.10	0.21	10.57	0.24	NS
Number of years of maternal education	9.36	0.02	9.76	0.43	10.21	0.21	9.85	0.25	NS

*M* mean, *n* number, *SE* standard error, *%* percentage, *A* not in survey, *B* wave 1 only, *C* wave 1 and 2, *D* participant of supplement wave 2b, *NS* not statistically significant, >statistically significantly different (*p* < 0.05)

^a^Native father/mother/grandfather/grandmother indicates the number and percentage of participants with a relative born in Israel

^b^At time of birth cohort member

^c^Chi-square and independent sample t tests were used to detect group differences

##### Jerusalem adolescent development study (JADs)

The Jerusalem Municipality routinely collected 8th grade school records (ages 13–14) from all state school students in the city from 1978 to 1988 (*n* = 21,449) [10]. In Israel, students attend the school nearest to their home and from the age of six, education is compulsory and free of charge. The Ministry of Education centrally determines study curriculum and examination content, and all children study the same core subjects for the same amount of time, facilitating comparison across schools.

All school grades ranged from 0 to 100 (excellent). Apart from school grades, teachers also rate their pupils on school behavior such as conduct, orderliness and motivation, on a scale ranging from 1 (low) to 6 (high). For each student, the final data archive included: (1) report card grades for academic core subjects (i.e., Hebrew, science, math and English); (2) grades for nonacademic subjects (i.e., physical education, music, arts and drawing and handicraft); and (3) teacher’ behavior ratings (i.e., conduct, orderliness and motivation).

##### Draft board assessment age 16–17

Between age 16 and 17, all Israeli Jewish citizens undergo a cognitive, behavioral, medical and psychiatric Draft board assessment to determine their eligibility for military service [12]. These assessments are centrally monitored for reliability and validity, and population-based norms are available [13, 14].

The cognitive assessment consists of four tests designed to measure IQ [15]. The IQ measures consist of the: (a) OTIS-R that measures the ability to understand and carry out verbal instructions; (b) Similarities-R that measures verbal abstraction and categorization; (c) Arithmetic-R that measures mathematical reasoning, concentration, and concept manipulation; and (d) Raven’s Progressive Matrices-R that measures nonverbal abstract reasoning and visual–spatial problem-solving abilities [15]. In males, a semi-structured interview assesses behavioral functioning including social functioning, individual autonomy, organizational ability, physical activity, and functioning in structured environments [13, 16]. Social functioning data were not available for females.

### National registry data

The national, continuously updated, registers STREAM data were linked to include the Ministry of Interior (including socio-demographic measures such as gender, year of birth, paternal country of origin, SES and marital status) [17–19], the Central Bureau of Statistics [to obtain neighborhood level socioeconomic status (SES) measure] [19, 20], and the Israeli National Psychiatric Hospitalization Case Registry [21]. The latter registry consists of medical records from medical and psychiatric assessments being done by Board-certified physicians and psychiatrists. Diagnoses are coded using ICD codes and all data are maintained in national databases.

## Procedures

Inclusion criteria were age between 34 and 44 years (born between 1964 and 1976) and raised in the inner-city of Jerusalem. People ever admitted to a psychiatric hospital were excluded.

Of the 7000 individuals appearing in both the birth cohort and the administrative cohort, 1000 were randomly sampled. At round one, participants aged 34–44 were approached to participate in a telephone survey until a number of 500 consented to participate (Wave 1, 2007–2008). One year later, the same individuals were approached to participate in wave 2 (2008–2009). A total of 80 % of those surveyed in wave 1 consented to participate in wave 2 and were re-assessed. To take an ±20 % anticipated attrition rate into account, this number was then supplemented by ascertaining consent and information from an additional 311 participants of the previously randomly selected sample (Wave 2 supplement), bringing up the sample to 811.

### Follow-up

**Fig. 1:**
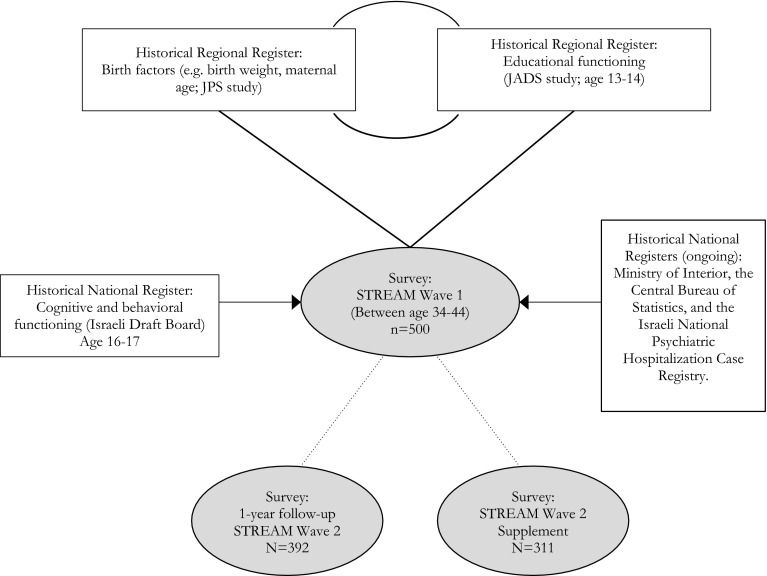
Linked data

At present, the ‘active’ prospective data collection of the STREAM study includes one large telephone survey conducted in 2007–2008 and a follow-up survey in 2008–2009. The next follow-up survey is planned for 2017–2018. Figure 1 presents a flowchart of the study design and linkage to other datasets.

### Ethics

Data management was done in accordance with established protocols for data protection (e.g., offline data storage and analysis). For the two telephone surveys, the STREAM team received ethical approval from the local Institutional Review Board to temporarily unmask anonymity (to establish telephone contact). Participants were explained the right of removal of their data, and data of those who chose to do so were excluded from the dataset. Permanently deleting all retraceable information and encrypting identifiers in the dataset available for research confidentiality was maintained.

## Sample characteristics

Table 1 presents characteristics of the remaining cohort members who were never contacted for follow-up (*n* = ~6000), and those participating in waves 1, 2, or both. Chi-square and independent *t* tests were conducted to examine group differences. A *p* value of <0.05 was considered statistically significant. Those who were not surveyed did not differ from participants in terms of age, gender, immigrant status of their parents and grandfather, SES and parental education.

Those never contacted, however, were more likely to have an older parent and a maternal grandmother who was born in Israel. Examination of between group differences on birth factors showed no statistically significant differences between groups (see Table 1).

## Results

### What has been measured?

Table 2 summarizes data collected through the telephone surveys. The first wave of the STREAM survey included questions about socio-demographics (marital status, religion, SES), resilience [22], social support [23], educational functioning, BMI, general health and mental health problems. For the latter, questions of the Mini International Neuropsychiatric Interview (MINI), a brief structured diagnostic interview for DSM-IV Axis I psychiatric disorders [24], were adapted to assess the continuum, onset and incidence of the psychiatric symptoms.

**Table 2:** Summary of STREAM survey measures

Core measures^a^	Question numbers^b^	Supplementary measures^a^	Question numbers^b^
Socio-demographics			
Marital status Religiosity Marital status Education	R1Q10, R1Q11, R1Q13-R1Q15 R3Q30-R3Q32, R4Q40	Occupation (and work tasks)	R2Q21m1-m2, R2Q22m1-m2, R2Q23m1-m3, R3Q27m1, R3Q28m1-m2, R3Q29m1
Physical (health) measures			
Subjective health (current/change last year)	R1Q1, R1Q2, R3Q1, R3Q2, R4Q34, R4Q35	Life style behavior (smoking/exercise) General health (e.g., diabetes/asthma/migraine) BMI	1Q12_1m1, R1Q12_2m1, R2Q20m1-m4, R2Q24m1, R3Q3_1, R3Q3_2, R3Q4m1–m3, R4Q37, R4Q38m1, R4Q39
Mental health			
Depression Anxiety (post-traumatic) stress Mania psychotic symptoms	R1Q3, R1Q5, R1Q6-R1Q8, R1Q6_1–R1Q6_5, R2Q2, R2Q3, R2Q3_1–R2Q3_4,R2Q4-R2Q16, R3Q5, R3Q6, R3Q10, R3Q10_1–R3Q10_4, R3Q11–R3Q23, R4Q5-R4Q19, R4Q19_1–R4Q19_4, RQ420-R4Q33, R4Q36	Autistic traits Obsessive compulsive and hoarding behavior	R2Q17-R2Q19, R3Q24–=R3Q26, R4Q1, R4Q1_1–R4Q1_5, R4Q2, R4Q3, R4Q4
(Candidate) protective factors			
Social support	R1Q4, R1Q4_1–RQ1_4, R3Q7, R3Q7_1–R3Q7_3	Flexibility/resilience Personality traits (e.g., Creativity)	R1Q9, R1Q9_1, R1Q9_2, R2Q1, R2Q1_1–R2Q1_6
			R3Q8, R3Q8_1, RQ3Q8_2, R3Q9, R3Q9_1–R3Q9_6

*SES* socioeconomic status

^a^Core measures = assessed at both waves. Supplementary measures part of one of the waves

^b^See supplementary Table 1. Annotated CRF with survey data variables

The second wave included the core questions of wave 1, but was expanded by questions on creativity, mania, psychotic symptoms, autistic traits [25], the cardinal features of OCD and hoarding [26], common health problems (e.g., allergy, diabetes), educational functioning and life style behaviors (e.g., smoking, exercise).

### What has it found? Key findings and publications

Table 3 presents first results on health outcomes of the first STREAM wave. Prevalence rates of health problems were comparable to recent World Mental Health Surveys [6] (e.g., the WHO reported prevalence rates of depression (5.7 %), post-traumatic stress disorder (PTSD) (1.5 %) and migraine (13.9 %) correspond to the rate of positive answers to questions about a lack of interest or pleasure of doing things (5.6 %), the experience of the traumatic event in dreams or flashback (1.5 %) and migraine (14.1 %)).

**Table 3:** Summary of core mental and general health measures of the STREAM survey

Baseline health measurements	Core measures Number of participants responding positive (%)
General health:	
2. Subjective health (not good/fair; *n* = 807)	85 (10.5)
Mental health	
Depression/anxiety: During the past 4 weeks, at least half of the days…	
1. A feeling of stress or anxiety (*n* = 804)	115 (14.3)
2. A feeling of being tense (a little or a lot more; *n* = 694)	112 (16.1)
3. Little interest or pleasure in doing things (*n* = 780)	44 (5.6)
4. A feeling of despondency, depression or hopelessness (*n* = 803)	27 (3.4)
5. A feeling of nervousness, anxiety, a feeling of being on edge or very concerned about different things (*n* = 700)	33 (14.0)
6. A feeling of embarrassment that people can see you (*n* = 699)	9 (1.3)
Mania (*n* = 702): a period in which the person was Extremely happy/full of ideas, or particularly nervous and annoyed (>2 weeks). During these times, (s)he was:	155 (19.1)
1. In trouble, in arguments or speaking fast because of it (mostly/always)	39 (4.8)
Psychotic symptoms: At least seldom, (s) he experienced:	
Unusual faith (*n* = 683)	112 (16.4)
The feeling of being spied on (*n* = 700)	68 (9.7)
The feeling of being deliberately harmed	73 (10.4)
Hallucinations (*n* = 692)	84 (12.1)
Voices (*n* = 170)	13 (1.9)
Trauma/PTSD Have you ever…	
8. Experienced or been a witness to a traumatic event involving death, danger of death or serious injury to you or someone close (*n* = 806)	373 (46.3)
During the past 4 weeks, at least half of the days…	
9. The experience of the traumatic event in dreams or flashback (369)	12 (1.5)
7. Ever: experienced an anxiety attack, a sudden feeling of fear or panic (*n* = 702)	82 (11.5)
Physical health (*n* = 810)	
Diabetes	5 (0.6)
Headaches/migraine	114 (14.1)
Cancer	5 (0.6)
Allergy/asthma	80 (9.9)

Over 90 % participants considered themselves to be of good health, irrespective of a relatively high number of individuals experiencing feelings of stress and anxiety on a daily basis (14 %).

Studies on data of the Jerusalem Municipality and Israeli Draft Board Registry thus far have predominantly focused on mental disorders, and schizophrenia in particular. For instance, school records have shown an association between poorer nonacademic school functioning and lower behavioral ratings with an earlier age of onset of schizophrenia [9]. Data of the Israeli Army Draft Board Registry alone pinpointed over 16 prediction markers of for schizophrenia, covering a range of domains including premorbid cognitive and social impairments [16, 27, 28], high population density [27] and low SES [29]. However, among others, studies utilizing data from the Israeli Army Draft Board also indicated associations between IQ and obesity in adolescence [30], and an advanced paternal age and autism [31].

## Strengths and weaknesses

As indicated above, the STREAM study provides a detailed overview of a broad range of health, adversity and resilience measures in a representative inner-city sample of the adult Jerusalem population. In contrast to most previous studies, in which data on social support and other resiliency factors are typically retrospectively extracted as part of a ‘not a priori designed instrument’, we actively probed for these factors, leading to a reliable dataset of potential resilience factors. Another major strength of the present cohort is the potential linkage to complete and continuously updated national registry sources, providing a wealth of additional information. Using national registries has the additional advantage of almost no attrition on certain aspects of the data (i.e., psychiatric hospitalizations, autism) unless a participant leaves the nation or dies. In addition, using a quasi-sequential design following up people born over 10 years, we were able to examine health outcomes over a one decade lifespan, and plan to continue future data collections. A limitation of the survey information is that it derives from self-reports using telephone surveys, which might be subject to report bias and limited the assessment to up to 30 questions per wave. In addition, our sample size of 811 is relatively small. This may affect the ability to detect causal associations and may limit the generalizability of findings from the STREAM study.

As more and more people migrate into cities, comparing our findings to other studies to health outcomes in city areas could be important. Equivalent studies have been done in Stockholm (The Stockholm Public Health Cohort [32]) and different city areas in Brasil (longitudinal study of adult health (ELSA-Brasil [33]) and it would be interesting to see how our results compare to these studies.

## Can I get hold of the data? Where can I find out more?

We welcome collaborative research that adds to or uses existing aspects of the STREAM data. The data pertaining to STREAM are held at the Department of Community Mental Health, University of Haifa, Israel. Data access is available by application to the STREAM principal investigators Stephen Z Levine at: levine.sz@gmail.com, or Jonathan Rabinowitz (jonathan.rabinowitz@biu.ac.il). Abraham Reichenberg and Eva Velthorst are collaborators on the project. Abraham Reichenberg instigated the cohort.

Key messages:

- STREAM is one of the first longitudinal Israeli cohort studies making use of national registry data and in-depth surveys, incorporating measures on symptom onset, course and subjective experience of commonly experienced health problems.
- Apart from measures on adverse psychosocial factors, STREAM provides a cohort to examine resilience to developing health problems and having a poor health and functional outcome.
- Over 90 % of the cohort considers themselves to be of good health, and the prevalence of mental health problems is comparable to that reported by WHO.

## Electronic supplementary material

Supplementary material 1 (DOCX 109 kb)
