# Supplementary material for: Study of resilience and environmental adversity in midlife health (STREAM)
Source: Soc Psychiatry Psychiatr Epidemiol. 2015 Oct 13;50(12):1915–22. doi: 10.1007/s00127-015-1126-y (PMC4654741; doi:10.1007/s00127-015-1126-y)
Supplement: Supplementary file 1 — Supplementary material 1 (DOCX 109 kb) [file 127_2015_1126_MOESM1_ESM.docx]

**Supplementary table 1. Annotated CRF with survey data variables**

| **Variable name** | **Variable label** | **Answer categories** |
| --- | --- | --- |
| R1Q1, R3Q1, R4Q34 | How would you assess your health in general? | - 1. Excellent - 2. Very good - 3. Good - 4. Fair/mediocre - 5. Not read/ don’t know |
| R1Q2, R3Q2, R4Q35 | In comparison to last year, is your health in general? | - 1. A lot better - 2. A little better - 3. Good - 4. Fair/ mediocre - 5. Not good - 6. Not read/ don’t know |
| R1Q3, R2Q2, R3Q5, R4Q36 | During the past four weeks, did you feel stress or anxiety? | - 1. Not at all - 2. For several days - 3. For about half the days - 4. For more than half the days - 5. Almost every day - 6. Not read/ don’t know/ refused to answer |
| R1Q4, R3Q7 | With whom of the following are you in contact, i.e. talk on the phone or meet, and how often? | - 1. Use - 2. No |
| R1Q4_1, R3Q7_1 | Parents, brothers, sisters | - 1. Yes, several times a week - 2. Yes, every day - 3. Yes, once a week - 4. Less than once a week - 5. Never - 6. Not read/ don’t know/ refused to answer |
| R1Q4_2, R3Q7_2 | Other relatives | - 1. Yes, several times a week - 2. Yes, every day - 3. Yes, once a week - 4. Less than once a week - 5. Never - 6. Not read/ don’t know/ refused to answer |
| R1Q4_3, R3Q7_3 | Friends | - 1. Yes, several times a week - 2. Yes, every day - 3. Yes, once a week - 4. Less than once a week - 5. Never - 6. Not read/ don’t know/ refused to answer |
| R1Q5, R3Q6 | In comparison to last year, how tense are you in general? | - 1. A lot more - 2. A little more - 3. At a similar level - 4. A little less - 5. A lot less - 6. Not read/ don’t know/ refused to answer |
| R1Q6, R2Q3, R3Q10, R4Q19 | During the past four weeks, were you disturbed by any of the following subjects? | - 1. Use - 2. No |
| R1Q6_1, R2Q3_1,  R3Q10_1, R4Q19_1 | Little interest or pleasure in doing things | - 1. Not at all - 2. For a few days - 3. For one-two weeks - 4. For more than two weeks - 5. Almost every day - 6. Not read/ don’t know/ refused to answer |
| R1Q6_2, R2Q3_2, R3Q10_2, R4Q19_2 | Feeling of despondency, depression or hopelessness | - 1. Not at all - 2. For a few days - 3. For one-two weeks - 4. For more than two weeks - 5. Almost every day - 6. Not read/ don’t know/ refused to answer |
| R1Q6_3, R2Q6_4, R3Q10_3 | A feeling of nervousness, anxiety, a feeling of being on edge or very concerned about different things | - 1. Not at all - 2. For a few days - 3. For one-two weeks - 4. For more than two weeks - 5. Almost every day - 6. Don’t know/ refused to answer |
| R1Q6_4, R2Q3_4, R3Q10_4 | A feeling of embarrassment that people can see you? | - 1. Not at all - 2. For a few days - 3. For one-two weeks - 4. For more than two weeks - 5. Almost every day - 6. Don’t know/ refused to answer |
| R1Q6_5, R2Q4, R3Q11, R4Q9 | An anxiety attack – a sudden feeling of fear or panic? | - 1. Not at all - 2. For a few days - 3. For one-two weeks - 4. For more than two weeks - 5. Almost every day - 6. Don’t know/ refused to answer |
| R1Q7, R2Q5, R3Q12 | Have you experienced or been a witness to a traumatic event involving death, danger of death or serious injury to you or someone close? | - 1. Yes, during the past month - 2. Yes, during the past year - 3. Yes, a year to three years ago - 4. Yes, more than three years ago - 5. Don’t know/ refuse to answer |
| R1Q8, R2Q6, R3Q13 | During the past four weeks, have you experienced the traumatic event in dreams or flashback? | - 1. Not at all - 2. For a few days - 3. For one-two weeks - 4. For more than two weeks - 5. Almost every day - 6. Don’t know/ refused to answer |
| R1Q9 | To what extent do the following sentences describe you? | - 1. Use - 2. No |
| R1Q9_1 | I can easily adapt to change. | - 1. To a very great extent - 2. To a great extent - 3. To a moderate extent - 4. To a minor extent - 5. Not at all - 6. Don’t know/ refused to answer |
| R1Q9_2 | I tend to come back to myself very quickly after illness or difficulty | - 1. To a very great extent - 2. To a great extent - 3. To a moderate extent - 4. To a minor extent - 5. Not at all - 6. Don’t know/ refused to answer |
| R1Q10, R3Q30 | Which is the last school you attended? Read 1-6). | - 1. Primary - 2. Partial high school - 3. Full high school - 4. Post high school without an academic degree - 5. Bachelors degree - 6. Masters degree and over |
| R1Q11 | How many years did you study? (including primary and high school). |  |
| R1Q12_1m1, R3Q3_1 | What is your height? (cm) | Refuse (999) |
| R1Q12_2m1, R2Q24m1, R3Q3_2 | What is your weight? (kg) | Refuse (999) |
| R1Q13 | Are you? (Read 1-5) | - 1. Single - 2. Married without children - 3. Married with children - 4. Divorced/widowed without children - 5. Divorced/widowed with children - 6. Don’t know/ refused to answer |
| R1Q14, R3Q31 | Are you? (Read 1-4) | - 1. Secular - 2. Traditional - 3. Religious - 4. Orthodox - 5. Non-Jewish |
| R1Q15, R3Q32, R4Q40 | The average income per household in Israel today is about NIS 8,000 Net. Is your total family income? (Read 1-5) | - 1. A lot below average - 2. Below average - 3. Around average - 4. Above average - 5. A lot above average - 6. Don’t know/ refused to answer |
| R2Q1, R3Q9 | I will read you a number of people’s features. For each one of these features, please tell me to what extent you think it characterizes you. | - 1. Use - 2.no |
| R2Q1_1, R3Q9_1 | Innovative | - 1. Not at all - 2. To a minor extent - 3. To a moderate extent - 4. To a great extent - 5. To a very great extent - 6. Don’t know/ refused to answer |
| R1Q2_2, R3Q9_2 | Creative | - 1. Not at all - 2. To a minor extent - 3. To a moderate extent - 4. To a great extent - 5. To a very great extent - 6. Don’t know/ refused to answer |
| R1Q2_3, R3Q9_3 | Conventional | - 1. Not at all - 2. To a minor extent - 3. To a moderate extent - 4. To a great extent - 5. To a very great extent - 6. Don’t know/ refused to answer |
| R1Q2_4, R3Q9_4 | Inquisitive | - 1. Not at all - 2. To a minor extent - 3. To a moderate extent - 4. To a great extent - 5. To a very great extent - 6. Don’t know/ refused to answer |
| R1Q2_5, R3Q9_5 | Artistic | - 1. Not at all - 2. To a minor extent - 3. To a moderate extent - 4. To a great extent - 5. To a very great extent - 6. Don’t know/ refused to answer |
| R1Q2_6, R3Q9_6 | Conservative | - 1. Not at all - 2. To a minor extent - 3. To a moderate extent - 4. To a great extent - 5. To a very great extent - 6. Don’t know/ refused to answer |
| R2Q7, R3Q14 | Was there ever a period in which you felt exceptionally happy, you were full of energy? Hyper? And needed less sleep than usual? (Read 1-5). | - 1. Not at all - 2. To a minor extent - 3. To a moderate extent - 4. To a great extent - 5. To a very great extent - 6. Don’t know/ refused to answer |
| R2Q8, R3Q15 | During those days when you felt happy and full of energy, did you get into trouble or did someone close to you say that you were behaving strangely and different from usual? (Read 1-4) | - 1. Never - 2. Sometimes - 3. Mostly - 4. Always - 5. Don’t know/ refused to answer |
| R2Q9, R3Q16 | During those days when you felt happy and full of energy, did you speak faster than usual, or did you feel that many, uncontrollable thoughts were racing in your head? (Read 1-4) | - 1. Never - 2. Sometimes - 3. Mostly - 4. Always - 5. Don’t know/ refused to answer |
| R2Q10, R3Q17 | Was there ever a time when you felt particularly nervous and annoyed? (Read 1-5) | - 1. Never - 2. For a few days - 3. For one or two weeks - 4. For more than two weeks - 5. Almost every day - 6. Don’t know/ refused to answer |
| R2Q11, R3Q18 | During these periods, were you involved in arguments, fights or did you shout at people? (Read 1-4) | - 1. Never - 2. Sometimes - 3. Mostly - 4. Always - 5. Don’t know/ refused to answer |
| R2Q12, R3Q19 | Have your relatives or friends ever considered your faith as strange or unusual? (Read 1-4) | - 1. Never - 2. Seldom - 3. Frequently - 4. Very frequently - 5. Don’t know/ refused to answer |
| R2Q13, R3Q20 | Have you ever felt that people were following you or spying on you? (Read 1-4) | - 1. Never - 2. Seldom - 3. Frequently - 4. Very frequently - 5. Don’t know/ refused to answer |
| R2Q14, R3Q21 | Have you ever felt that people are deliberately acting against you and want to harm you or your interests? (Read 1-4) | - 1. Never - 2. Seldom - 3. Frequently - 4. Very frequently - 5. Don’t know/ refused to answer |
| R2Q15, R3Q22 | Did you ever hear or see things that other people cannot see or hear? (Read 1-4) | - 1. Never - 2. Seldom - 3. Frequently - 4. Very frequently - 5. Don’t know/ refused to answer |
| R2Q16, R3Q23 | Have you ever heard voices saying all sorts of things when there was nobody else around?(Read 1-4). | - 1. Never - 2. Seldom - 3. Frequently - 4. Very frequently - 5. Don’t know/ refused to answer |
| R2Q17, R3Q24 | To what extent do you have difficulty in throwing out or giving away old objects that other people part from easily? (Read 1-5). | - 1. I never have such difficulty - 2. I usually don’t have such difficulty - 3. I sometimes have such difficulty - 4. I usually have such difficulty - 5. I always have such difficulty - 6. Don’t know/ refused to answer |
| R2Q18, R3Q25 | To what extent do you have difficulty in using rooms in your house because of disorder or clutter of objects? (Read 1-5) | - 1. I never have such difficulty - 2. I usually don’t have such difficulty - 3. I sometimes have such difficulty - 4. I usually have such difficulty - 5. I always have such difficulty - 6. Don’t know/ refused to answer |
| R2Q19, R3Q26 | To what extent do you experience distress in your daily life due to the difficulty to throw out objects or the accumulation of objects? (Read 1-5). | - 1. I never feel distressed - 2. I usually don’t feel distressed - 3. I sometimes feel distressed - 4. I usually feel distressed - 5. I always feel distressed - 6. Don’t know/ refused to answer |
| R2Q20m1-m4, R3Q4m1-m3 | Do you suffer from any of the following? (You can mark more than one item). | - 1. Allergy or asthma - 2. Diabetes - 3. Heart problems - 4. Headaches - 5. Cancer - 6. High blood pressure - 7. Low blood pressure - 8. Back problems - 9. Healthy - 10. Don’t know/ refused to answer |
| R2Q21m1-m2, R3Q27m1 | What is your occupation? | -open- |
| R2Q22m1-m2, R3Q28m1-m2 | What is the definition of your position in your work place? | -open- |
| R2Q23m1-m3, R3Q29m1 | Please describe the main activities you perform at your work place. | -open- |
| R3Q8 | To what extent do the following sentences describe you? | - 1. Use - 2. No |
| R3Q8_1 | I can easily adapt to change | - 1. To a very great extent - 2. To a great extent - 3. To a moderate extent - 4. To a minor extent - 5. Not at all - 6. Don’t know/ refuse to answer |
| RQ3Q8_2 | I tend to come back to myself very quickly after illness or difficulty | - 1. To a very great extent - 2. To a great extent - 3. To a moderate extent - 4. To a minor extent - 5. Not at all - 6. Don’t know/ refuse to answer |
| R4Q1 | Following are a number of statements. Please tell me to what extent you agree with each one: (Read the answer scale). | - 1. Use - 2. No |
| R4Q1_1 | I usually concentrate more on the overall picture rather than on the small details. | - 1. Highly agree - 2. Agree - 3. Partially agree - 4. Do not agree so much - 5. Do not agree at all - 6. Don’t know/ refuse to answer |
| R4Q1_2 | I easily succeed in doing several things at once. | - 1. Highly agree - 2. Agree - 3. Partially agree - 4. Do not agree so much - 5. Do not agree at all - 6. Don’t know/ refuse to answer |
| R4Q1_3 | I easily recognize if someone I am talking to is bored. | - 1. Highly agree - 2. Agree - 3. Partially agree - 4. Do not agree so much - 5. Do not agree at all - 6. Don’t know/ refuse to answer |
| R4Q1_4 | When I read a story, I insist on understanding the characters’ intentions. | - 1. Highly agree - 2. Agree - 3. Partially agree - 4. Do not agree so much - 5. Do not agree at all - 6. Don’t know/ refuse to answer |
| R4Q1_5 | It is easy for me to understand what a person is thinking or feeling just by looking at his face. | - 1. Highly agree - 2. Agree - 3. Partially agree - 4. Do not agree so much - 5. Do not agree at all - 6. Don’t know/ refuse to answer |
| R4Q2 | To what extent do you have unwanted, unpleasant or embarrassing thoughts, impulses or fantasies? For example, thinking that you are dirty, that you are sick with a contagious disease, that you have germs, or fear of behaving impulsively and harming someone? | - 1. I never have - 2. I usually don’t have - 3. I sometimes have - 4. I usually have - 5. I always have - 6. Don’t know/ refuse to answer |
| R4Q3 | To what extent do you repeat things over again, without being able to stop doing them? For example, washing your hands all the time, counting things or checking things again and again, arranging things again and again, or other things. (Read 1-5) | - 1. Never - 2. Not usually - 3. Sometimes - 4. Usually - 5. Always - 6. Don’t know/ refuse to answer |
| R4Q4 | To what extent did you feel that the thoughts or behavior you indicated significantly disturbed you in your daily life, at work, in your social life, or in personal relationships? (Read 1-5) | - 1. Never - 2. Not usually - 3. Sometimes - 4. Usually - 5. Always - 6. Don’t know/ refuse to answer |
| R4Q5 | What was the longest period of time you felt stress or anxiety almost every day? (Read 1-5) | - 1. Never - 2. Less than a week - 3. Between a week to a month - 4. More than a month but less than six months - 5. About six months or more - 6. Don’t know/ refuse to answer |
| R4Q6 | How old were you when it first happened? | -open- |
| R4Q7 | Did you have difficulty in controlling the feeling of stress or anxiety? (Read 1-5) | - 1. Not at all - 2. To a minor extent - 3. To a moderate extent - 4. To a great extent - 5. To a very great extent - 6. Don’t know/ refuse to answer |
| R4Q8 | Did you feel stress or anxiety during the last four weeks? (Read 1-5) | - 1. Not at all - 2. For several days - 3. About half of the days - 4. More than half of the days - 5. Almost every day - 6. Don’t know/ refuse to answer |
| R4Q9 | During the last four weeks, did you experience an anxiety attack? A sudden feeling of fear or panic? (Read 1-5) | - 1. Not at all - 2. For several days - 3. About half of the days - 4. More than half of the days - 5. Almost every day - 6. Don’t know/ refuse to answer |
| R4Q10 | Have you experienced or been in a traumatic event involving death, danger of death or serious injury to you or someone close? (Read 1-5) | - 1. Yes, during the past month - 2. Yes, during the past year - 3. Yes, a year / three years ago - 4. Yes, more than three years ago - 5. No   6. Don’t know/ refuse to answer |
| R4Q11 | How old where you when this event occurred | –open- |
| R4Q12 | Have you ever experienced the traumatic event in dreams or ‘flashbacks’ (Read 1-5) | - 1. Not at all - 2. For a few days - 3. For about a week to two weeks - 4. For more than two weeks - 5. Almost every day - 6. Don’t know/ refuse to answer |
| R4Q13 | How old were you when you experienced the dream or ‘flashback’ for the first time? | - -open- |
| R4Q14 | During the past four weeks, have you experienced the traumatic event in dreams or ‘flashback’?? (Read 1-5) | - 1. Not at all - 2. For a few days - 3. For about a week to two weeks - 4. For more than two weeks - 5. Almost every day   6. Don’t know/ refuse to answer |
| R4Q15 | What was the longest period of time when you felt a sense of despondency, depression, or hopelessness most hours of the day? (Read 1-5) | - 1. Never - 2. A few days - 3. One to two weeks - 4. More than two weeks - 5. Almost every day - 6. Don’t know/ refuse to answer |
| R4Q16 | How old were you when you experienced these feelings for the first time? | -open- |
| R4Q17 | What was the longest period of time when you had no interest in or didn’t enjoy doing things? (Read 1-5) | - 1. Never - 2. A few days - 3. One to two weeks - 4. More than two weeks - 5. Almost every day   6. Don’t know/ refuse to answer |
| R4Q18 | How old were you when you first experienced these feelings? | -open- |
| R4Q20 | Was there ever a period in which you felt exceptionally happy, you were full of energy, ‘hyper’ and needed less sleep than usual? (Read 1-5) | - 1. Not at all - 2. For a few days - 3. For about a week to two weeks - 4. For more than two weeks - 5. Almost every day - 6. Don’t know/ refuse to answer |
| R4Q21 | How old were you when for the first time there was a period when you felt exceptionally happy, you were full of energy, ‘hyper’ and needed less sleep than usual? | - -open- |
| R4Q22 | During those days when you felt happy and full of energy, did you get into trouble or did someone close to you say that you were behaving strangely and different from usual? (Read 1-4) | - 1. Never - 2. Sometimes - 3. Mostly - 4. Always - 5. Don’t know/ refuse to answer |
| R4Q23 | During those days when you felt happy and full of energy, did you speak faster than usual, or did you feel that many, uncontrollable thoughts were racing in your head? (Read 1-4) | - 1. Never - 2. Sometimes - 3. Mostly - 4. Always   5. Don’t know/ refuse to answer |
| R4Q24 | Was there ever a time when you felt particularly nervous and annoyed? (Read 1-5) | - 1. Never - 2. For a few days - 3. For one to two weeks - 4. For more than two weeks - 5. Almost every day - 6. Don’t know/ refuse to answer |
| R4Q25 | How old were you when you first felt like that? | - -open- |
| R4Q26 | During these periods, were you involved in arguments, fights or did you shout at people? (Read 1-4) | - 1. Never - 2. Sometimes - 3. Mostly - 4. Always - 5. Don’t know/ refuse to answer |
| R4Q27 | Have your relatives or friends ever considered your faith as strange or unusual? (Read 1-4) | - 1. Never - 2. Seldom - 3. Frequently - 4. Very frequently   5. Don’t know/ refuse to answer |
| R4Q28 | Have you ever felt that people were following you or spying on you? (Read 1-4) | - 1. Never - 2. Seldom - 3. Frequently - 4. Very frequently - 5. Don’t know/ refuse to answer |
| R4Q29 | How old were you when you first felt that people were following you or spying on you? | - -open- |
| R4Q30 | Have you ever felt that people are deliberately acting against you and want to harm you or your interests? (Read 1-4) | - 1. Never - 2. Seldom - 3. Frequently - 4. Very frequently   5. Don’t know/ refuse to answer |
| R4Q31 | Did you ever hear or see things that other people cannot see or hear? (Read 1-4) | - 1. Never - 2. Seldom - 3. Frequently - 4. Very frequently - 5. Don’t know/ refuse to answer |
| RQ32 | How old were you the first time you heard or saw things that others cannot see or hear? | - - open - |
| RQ33 | Have you ever heard voices saying all sorts of things when there was nobody else around?(Read 1-4) | - 1. Never - 2. Seldom - 3. Frequently - 4. Very frequently - 5. Don’t know/ refuse to answer |
| R4Q37 | During the past year, have you been engaged in any sports activity, each activity lasting 30 minutes continuously? If so, how often do you engage in this activity? | - 1. I engage in regular sports activity 3 or more times a week - 2. I engage in regular sports activity twice a week - 3. I engage in regular sports activity once a week - 4. I engage in regular sports activity about three times a month - 5. Once or twice a month or sometimes occasionally - 6. I engage in regular sports activity only part of the year (e.g. swimming in summer and running in winter) - 7. I sometimes engage in sports activity, but not on a regular basis - 8. I don’t engage in any sports activity |
| R4Q38m1 | What is the type of sports activity that you do regularly, not less than 30 minutes continuously? (If more than one activity, mark only the main one) | - 1. Any type of exercise done at home - 2. Any type of exercise done outside the home (classes, club, Spinning, Pilates, etc.) - 3. Running – Jogging - 4. Swimming - 5. Tennis - 6. Ball games (soccer, basket ball, volley ball, etc.) - 7. Walking, hiking - 8. Any type of dancing - 9. Folk dancing - 10. Field bike riding - 11. Cycling - 12. Gym/ fitness devices - 13. Judo, Karate (any types of hand-to-hand combat) - 14. Sailing, surfing and beach sports - 15. Other |
| R4Q39 | Do you smoke or did you smoke in the past? | - 1. I don’t smoke - 2. I smoked in the past but I stopped - 3. I smoke - 4. Refuse to answer |
